# First cases of *Burkholderia cenocepacia* IIIA neonatal sepsis in Central African Republic

**DOI:** 10.11604/pamj.2020.36.330.24512

**Published:** 2020-08-24

**Authors:** Thierry Frank, Julita Gil-Cuesta, Jean Robert Mbecko, Hugues Sanke, Chantal Lakis, Anne Le Flèche-Matéos, Alain Berlioz-Arthaud

**Affiliations:** 1 Institut Pasteur de Bangui, Unité de Bactériologie, Laboratoire de Biologie Médicale, Bangui, République Centrafricaine,; 2 Médecins Sans Frontières, Operational Center, Brussels, Belgique,; 3 Institut Pasteur, Cellule d´Intervention Biologique d´Urgence (CIBU), Paris, France

**Keywords:** *Burkholderia cenocepacia*, sepsis, Central African Republic

## Abstract

Bacteria of the Burkholderia cepacia complex cause frequent infections in immunocompromised and hospitalized patients, with a significant mortality rate. Phenotypic identification of those bacteria is difficult and therefore rarely reported from developing countries. This study presents the first ever reported case series of Burkholderia cenocepacia neonatal sepsis in Central African Republic. It demonstrates the superiority of molecular methods to accurately identify B. cenocepacia IIIA species compared to the phenotypic methods.

## Introduction

*Burkholderia cenocepacia* is a species of Gram-negative bacteria that is widespread in the environment, and is also an opportunistic pathogen causing chronic lung infections in patients with cystic fibrosis as well as other immunocompromised patients [1]. This pathogen is frequently associated with reduced survival and higher risk of developing fatal cepacia syndrome [2]. Taxonomic studies allowed designation of binomial species names for clinically isolated *Burkholderia*, which are now referred to as species of the *Burkholderia cepacia* complex (Bcc), composed of at least 17 species, including *B. cepacia* [3] and *B. cenocepacia*. Using *recA* sequencing and multilocus sequence typing, *B. cenocepacia* isolates can be subdivided into four distinct lineages, IIIA, IIIB, IIIC, and IIID. To date the majority of clinical isolates, belong to the IIIA, IIIB, and IIID lineages [4]. Bcc bacteria, particularly *B. cenocepacia*, are naturally resistant to different classes of antibiotics used in clinical practice and their pathogenicity is promoted by several virulence determinants. These characteristics, together with the ability to adapt to environmental changes, make the treatment of *B. cenocepacia* infections particularly challenging. Here, we describe the first cases of bacteremia caused by *B. cenocepacia* in Central African Republic (CAR).

## Methods

From 07/01/2018 to 08/02/2018, five newborns were born in a maternity of Bangui, CAR through vaginal delivery (four) and caesarean section (one). Three were males and two females. They were admitted at the maternity neonatal unit due to infection risk (two neonates), severe asphyxia (one) or prematurity (one). During the admission, they were all suspected of sepsis, presenting fever which was treated with ampicillin and gentamicin (first line antibiotic therapy according to the clinical protocol). They were all changed to second line with cefotaxime, after at least 48 hours of first line. Four of the neonates were discharged clinically well, while the fifth one died. Blood samples were collected from symptomatic children after 48 hours of not responding to first line and submitted to the Bacteriology Laboratory of the Pasteur Institute of Bangui. All samples were tested including Gram staining, culture on BHI (Brain Heart Infusion) media, oxidase test and API 20 NE strips (bioMérieux, Marcy l'Étoile, France). Antibiotic susceptibility was determined by the disc diffusion method (Bio-Rad, Marnes-la-Coquette, France) on Mueller-Hinton agar and interpreted according to the 2017 recommendations of the CA-SFM (Comité de l'Antibiogramme de la Société Française de Microbiologie). Total DNA was prepared from bacterial cultures by using the Promega Genomic DNA purification kit (Promega, Madison, WI, USA). A whole-genome shotgun sequencing experiment and assembly of the five stains, named 18-0020, 18-0021, 18-0022, 18-0023 and 18-0024, was performed using the Next Generation Sequencing (NGS) technique (Illumina Miseq). The Multilocus Sequence Analysis (MLSA) scheme using a concatenate of seven housekeeping gene portions (atpD, gltB, gyrB, recA, lepA, phaC, trpB) [5] yielded an unrooted tree displaying 20 known species of the Bcc and Burkholderia fungorum was used as an outgroup. Sequences of the 20 different species are available on the Bcc PubMLST database (https: //pubmlst.org/bcc). A total of 2773 bp for MLSA scheme were determined for each species. Related sequences were downloaded, compared and phylogenetic tree was generated with CLC Main Workbench 7.8.1 software (Qiagen), using the neighbour-joining (NJ) algorithm [6].

## Results

**Figure 1:**
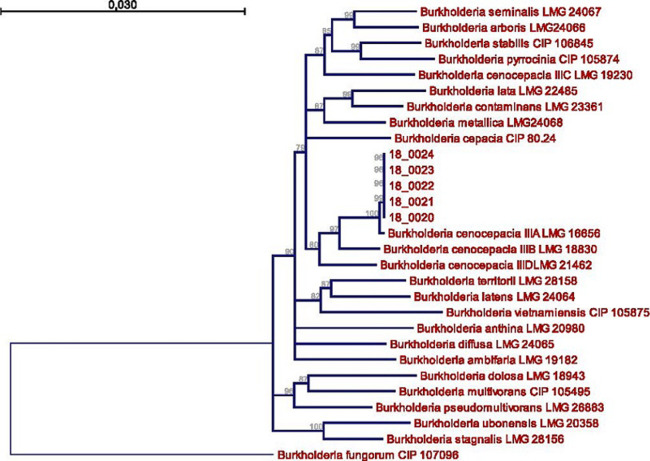
neighbour-joining unrooted tree, based on MLSA, including seven genes (atpD, gltB, gyrB, recA, lepA, phaC, trpB); all sequences came from types strains: bootstrap values > 75% (based on 1000 replicates) are indicated by thick lines; bar 0.030 substitutions per nucleotide position

All samples were positive for a Gram-negative bacillus identified as *Burkholderia cepacia* by API 20 NE. In addition, the strains were resistant to piperacillin, ticarcillin-clavulanate, ceftazidime, cefepime, imipenem and gentamicin but three isolates remained susceptible to cotrimoxazole. As *B. cepacia* had never been previously isolated from blood culture in CAR, we chose to identify the strains by using molecular typing. The NJ tree derived from atpD, gltB, gyrB, recA, lepA, phaC, trpB genes sequences showed the five isolates belong to *Burkholderia cenocepacia* IIIA. All five isolates were 100% identical for each internal portion of selected housekeeping genes (Figure 1).

## Discussion

These molecular results are not surprising, because species of Bcc can express common multiple chemical characters not discriminable by the API 20 NE [7]. Furthermore, *B. cenocepacia* IIIA encodes several factors involved in the pathogenesis of the bacteria, including surface polysaccharides, adhesins, flagella, lipase, siderophores, efflux pumps, quorum-sensing systems and metalloproteases, among others. The multiple virulence factors encoded by *B. cenocepacia* confer to this bacteria the capacity to invade epithelial cells, to degrade immunoglobulins, to coordinate cellular activities, to resist reactive oxygen, nitrogen, serum complement, antimicrobial peptides, phagocytosis and finally to invade the host. In addition, *B. cenocepacia* IIIA includes a group of strains that have been causing severe infections reported in Canadian, UK and European populations [8].

## Conclusion

In conclusion, we present here the first ever reported case series of Bcc neonatal sepsis in CAR, occurring during admission in a maternity of Bangui. The present study also demonstrates the application of a molecular method based on the MLSA scheme to accurately identify *B. cenocepacia* IIIA species as the phenotypic methods based on biochemical analysis proved not to be appropriate for the identification of Bcc. Therefore, we show the usefulness of a molecular technique in microbiology laboratories to process specimens from patients with Bcc infections.

### What is known about this topic

- Bacteria from the Burkholderia cepacia complex (Bcc) are serious opportunistic pathogens in immunocompromised and cystic fibrosis patients and has also been linked to healthcare-associated outbreaks, mainly in developed countries;
- Biochemical analysis of species among the Bcc is poorly discriminant and often leads to misidentification.

### What this study adds

- This study describes an occurrence of B. cenocepacia sepsis in newborns in a maternity of Bangui (CAR): pediatric cases are rarely reported, even more in developing countries, our series is the first ever reported in Bangui;
- We confirm the efficiency of multilocus sequence analysis (MLSA) for accurate identification of the B. cenocepacia species.
